# Breastfeeding Practices, Insomnia Symptoms, Anxiety, and Depression Among Lebanese Mothers: A Cross‐Sectional Study

**DOI:** 10.1002/fsn3.71830

**Published:** 2026-05-22

**Authors:** Paula Hage Boutros, Nour Chamma, Hala Sacre, Pascale Salameh, Chadia Haddad, Cosette Fakih El Khoury, Joanne Karam, Rana Rizk, Bahia Abdallah

**Affiliations:** ^1^ d'Épidémiologie Clinique et de Toxicologie‐Liban (INSPECT‐LB) Institut National de Santé Publique Beirut Lebanon; ^2^ Department of Nutrition and Dietetics Modern University for Business and Science Beirut Lebanon; ^3^ Department of Nutrition and Food Science, School of Arts and Sciences Lebanese American University Byblos Lebanon; ^4^ Faculty of Pharmacy Lebanese University Hadat Lebanon; ^5^ Gilbert and Rose‐Marie Chagoury School of Medicine Lebanese American University Beirut Lebanon; ^6^ Department of Primary Care and Population Health University of Nicosia Medical School Nicosia Cyprus; ^7^ Research Department Psychiatric Hospital of the Cross Jal Eddib Lebanon; ^8^ Alice Ramez Chagoury School of Nursing Lebanese American University Byblos Lebanon

**Keywords:** anxiety, breastfeeding, depression, insomnia, Lebanon, mental health

## Abstract

The postpartum period is marked by significant physiological and psychological changes, placing mothers at heightened risk for mental health disturbances such as anxiety, depression, and insomnia. While breastfeeding is widely recognized for its physical and emotional benefits, its association with maternal mental health outcomes in low‐ and middle‐income countries, particularly Lebanon, remains underexplored. This study aimed to assess the relationship between breastfeeding practices (exclusive breastfeeding at 6 months and breastfeeding continuation at 12 and 24 months) and insomnia, anxiety, and depression among Lebanese mothers, adjusting for sociodemographic, pregnancy and delivery, dietary, and lifestyle variables. A cross‐sectional online survey was administered in 2023–2024 to Lebanese mothers who had given birth between 2018 and 2023. Participants (*n* = 305) completed validated tools including the Patient Health Questionnaire (PHQ‐4) for anxiety and depression, the Women's Health Initiative Insomnia Rating Scale (WHIIRS), and questionnaires on breastfeeding practices, sociodemographic status, dietary habits, and household food security. Statistical analyses examined associations between breastfeeding behaviors and mental health outcomes. Exclusive breastfeeding at 6 months was not associated with maternal insomnia, anxiety, or depression. In contrast, breastfeeding continuation at 12–24 months was positively associated with insomnia (aOR = 2.15). Beyond breastfeeding indicators, insomnia was also associated with anxiety (aOR = 2.42) and with lifestyle‐related factors, including lower adherence to the Mediterranean diet (aOR = 0.80). Anxiety was associated with perinatal factors (shorter pregnancy duration: aOR = 0.89; absence of immediate skin‐to‐skin contact: aOR = 0.49), family characteristics (higher parity: aOR = 1.70), lifestyle factors (tobacco use: aOR = 2.69), and co‐occurring mental health symptoms (depression: aOR = 19.79; insomnia: aOR = 2.36). Depression was primarily associated with food insecurity (aOR = 1.15) and anxiety (aOR = 20.00), and was inversely associated with alcohol consumption (aOR = 0.37). Overall, anxiety and depression were more consistently related to social, behavioral, and socioeconomic determinants than to breastfeeding practices. These results underscore the importance of routine perinatal mental health screening, in line with current recommendations, and of interventions targeting modifiable factors such as tobacco use and food insecurity, rather than framing breastfeeding itself as a risk factor for maternal mental health difficulties.

## Graphical abstract

Breastfeeding practices were not major determinants of maternal mental health. Exclusive breastfeeding showed no association, while continued breastfeeding was linked to higher insomnia. Mental health outcomes were more strongly influenced by lifestyle and socioeconomic factors.

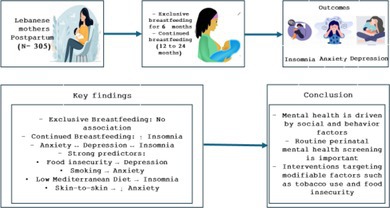

## 1 Introduction

Breastfeeding (BF) is the optimal infant feeding method, as breast milk provides essential nutrients for a baby's growth and development during the first 6 months of life, adjusting its composition to meet changing nutritional needs (Chowdhury et al. 2015). It contains carbohydrates primarily as lactose (the main energy source) and human milk oligosaccharides that promote beneficial gut microbiota and immune maturation; high‐quality proteins (whey and casein fractions) supplying essential amino acids and bioactive factors such as lactoferrin and immunoglobulins; lipids predominantly as triacylglycerols, including essential fatty acids and long‐chain polyunsaturated fatty acids (e.g., DHA and ARA) critical for neurodevelopment; a full complement of fat‐ and water‐soluble vitamins; and highly bioavailable minerals such as calcium, iron, zinc, and iodine, all delivered in a water‐rich matrix that ensures hydration and physiological balance (Ballard and Morrow 2013).

Due to its unique composition, BF milk significantly reduces the risk of infant mortality and early childhood allergic diseases (Lodge et al. 2015; Sankar et al. 2015), supports better cognitive outcomes (Brion et al. 2011), and is associated with a healthier metabolic profile in adulthood (Horta et al. 2015). Besides its health merits, BF is cost‐effective and reduces healthcare costs by lowering the incidence of illnesses (Quesada et al. 2020). For mothers, BF reduces the risk of breast and ovarian cancers (Chowdhury et al. 2015), promotes hormonal regulation, and facilitates postpartum amenorrhea (Chowdhury et al. 2015; Modak et al. 2023). Both the World Health Organization (WHO) and the United Nations International Children's Emergency Fund (UNICEF) recommend early initiation of BF, exclusive BF during the first 6 months, and continued BF until 24 months and beyond (Sankar et al. 2015; UNICEF, 2025; World Health Organization, 2025).

In addition to BF's physical health benefits, emerging evidence suggests its potential role in supporting maternal mental health. BF has been linked to reductions in stress hormones, enhanced self‐efficacy, enhanced maternal mood, and improved mother‐infant bonding (Tucker and O'Malley 2022). However, its association with maternal insomnia, anxiety, and depression remains poorly understood and requires further investigation. Postpartum sleep disturbances are common and often characterized by reduced duration and poor quality across the first postpartum year, even as sleep gradually improves (Witkowska‐Zimny et al. 2024). Studies comparing BF and bottle‐feeding yielded mixed results. While breastfeeding has been associated with slightly longer nighttime sleep (Srimoragot et al. 2022) and better subjective sleep quality (Newman et al. 2023), most evidence indicates similar total sleep and sleep quality across feeding methods despite more night awakenings among breastfeeding mothers (Manková et al. 2023; Mariman et al. 2024). Proposed mechanisms include the presence of sleep‐promoting components in human milk, such as melatonin, tryptophan, and specific nucleotides, as well as hormonal effects in mothers, notably prolactin and oxytocin release during nighttime feeds, which facilitate relaxation and rapid return to sleep (Bilodeau et al. 2025).

Higher formula use early postpartum is associated with shorter maternal sleep (Ruan et al. 2022). Recent longitudinal work emphasizes that frequent night feedings, rather than feeding method per se, are the main disruptor of maternal sleep (Astbury et al. 2022; Witkowska‐Zimny et al. 2024). Interpretation is further complicated by methodological heterogeneity (objective vs. subjective measures, variable timepoints) and the bidirectional overlap between sleep problems and mood symptoms, with postpartum insomnia/poor sleep predicting higher depression and anxiety—and depressed mood later predicting ongoing sleep disturbance (Gauld et al. 2024; Newman et al. 2023; Okun and Lac 2023; Rudzik et al. 2023).

Postpartum anxiety (PPA) has increasingly been implicated in suboptimal breastfeeding outcomes, with higher anxiety linked to delayed initiation, shorter duration, increased feeding difficulties, reduced exclusivity, and stress‐related alterations in human milk composition that may affect lactation and infant outcomes (Feldman et al. 2025; Hoff et al. 2019; Matyas et al. 2024; Nagel et al. 2022; Neupane et al. 2025; Rosen‐Carole et al. 2024). The relationship between breastfeeding and postpartum depression (PPD) is similarly complex and appears bidirectional. Increased duration and intensity of BF have been associated with fewer depressive symptoms: a recent systematic review and meta‐analysis found that mothers who did not exclusively breastfeed had higher odds of PPD compared with those who exclusively breastfed: OR = 1.89 (95% CI 1.50–2.39) (Alimi et al. 2022). This finding is also especially true among women with higher education levels (Xia et al. 2022). Conversely, depression can undermine breastfeeding self‐efficacy and persistence (Ahmadinezhad et al. 2024; Bondade et al. 2025).

Insomnia, anxiety, and depression are closely interrelated during the postpartum period, with each condition potentially exacerbating the others (Swanson et al. 2020). Insomnia, in particular, may predict future depression and anxiety, often through nocturnal rumination about infant health, resulting in chronic sleep disturbances (Swanson et al. 2020). Conversely, anxiety and depression can also lead to insomnia through several interconnected mechanisms, including cognitive hyperarousal (such as excessive worry about the inability to sleep), delayed sleep onset, and daytime impairment (Dørheim et al. 2012; Leistikow and Smith 2024). Furthermore, both anxiety and depression are associated with elevated inflammatory markers and disruptions in circadian rhythm, which further compromise sleep quality and regulation (Hall et al. 2022; Silva‐Fernandes et al. 2024). In this context, PPA and PPD are particularly intertwined, as they frequently co‐occur and share overlapping risk factors and symptoms, including mood dysregulation, sleep disturbances, and impaired daily functioning (Biaggi et al. 2016; Falah‐Hassani et al. 2016).

Thus, evidence linking breastfeeding to maternal mental health remains inconsistent, in part because the relationship appears context‐dependent, heterogeneous, and potentially bidirectional. There is a possibility that breastfeeding difficulties or unmet expectations may coincide with worse mental health, complicating causal interpretation (Yuen et al. 2022), or better health outcomes (Alimi et al. 2022). Longitudinal work further supports bidirectionality: perinatal depressive symptoms can predict shorter or less intense breastfeeding, while breastfeeding patterns may also precede later changes in depressive symptoms, depending on timing and analytic approach (Zhu et al. 2023). For sleep‐related outcomes, findings are similarly mixed across cohorts and measurement strategies, with some studies reporting little to no difference in sleep quality or duration by breastfeeding status and others observing distinct sleep disruptions (Astbury et al. 2022; Smith and Forrester 2021).

In Lebanon, concerns about maternal mental health are compounded by high rates of PPA and PPD; reports indicate that Lebanese mothers experience moderate postpartum insomnia, especially with their second or third baby (Hobeika et al. 2023). The bidirectional relationship between these three conditions is well‐established, with each increasing the risk of the other (Leistikow and Smith 2024; Okun and Lac 2023). Furthermore, maternal insomnia and sleep deprivation can adversely affect BF practices. Poor sleep quality predicts lower milk volume and production: disrupted or insufficient sleep may impair lactation physiology, potentially via effects on lactation‐related hormones such as prolactin and oxytocin, which are regulated by circadian rhythms and can be sensitive to sleep loss, as well as through increased stress and fatigue that adversely affect overall milk production (Carrega et al. 2020; Islam et al. 2021); moreover, sleep deprivation undermines BF self‐efficacy, and increases the likelihood of early BF cessation (Ahmadinezhad et al. 2024). These effects may help explain the suboptimal BF practices in Lebanon. The 2023–2024 Lebanon Integrated Micronutrient, Anthropometry, and Child Development Survey (LIMA) found that while over 80% of children under two were ever breastfed, only 60% initiated BF within the first hour after delivery, and more concerning, just 23% of infants under 6 months were exclusively breastfed (Ministry of Public Health (Lebanon) et al. 2024).

Against the backdrop of mixed and methodologically diverse evidence, the central knowledge gap is not whether breastfeeding is associated with a better or worse maternal mental health, but under what conditions, and net of key social and behavioral determinants, breastfeeding exclusivity and continuation are associated with specific maternal mental health outcomes, which this study directly investigates. Moreover, in the Lebanese context, this study examines associations between breastfeeding practices and maternal insomnia, anxiety, and depression among Lebanese mothers in the postpartum period, adjusting for sociodemographic variables, pregnancy and delivery factors, dietary habits, household food security, and lifestyle practices. The aim is to inform interventions that promote both maternal mental health and successful breastfeeding.

## 2 Methods

### 2.1 Design

This cross‐sectional study was conducted among a sample of Lebanese adult mothers using an online survey on Google Forms. Data were collected from December 3, 2023, to March 16, 2024. Participants were recruited via social media (targeted Facebook and WhatsApp groups) using a snowball technique to enhance social, educational, and cultural diversity and reach optimal geographical representativity within Lebanese regions, as much as possible given the non‐random nature of the sample.

### 2.2 Study Population

Eligible participants were women aged 18 years and older, residing in Lebanon, who had given birth between 2018 and 2023, and were willing to complete the online survey. Exclusion criteria included women residing in refugee camps, Lebanese women living abroad, those unable to complete the survey due to technical or other limitations, and women who had not given birth prior to 2018.

### 2.3 Measures

The survey was available in both English and Arabic, allowing participants to select their preferred language. The validated measures were taken from publicly available English versions, and the Arabic wording followed established translation procedures in line with WHO best practices (World Health Organization 2010).

The first section of the questionnaire assessed sociodemographic, lifestyle, and employment characteristics, including age, marital status, number of children, educational level, place of residence, area of living, monthly household income, smoking habits, alcohol consumption, intake of dietary supplements, and current occupation. In addition, household crowding was evaluated using the crowding index, which is calculated by dividing the number of people living in a dwelling by the number of rooms, excluding kitchens and bathrooms.

This section also comprised pregnancy‐related questions (height, pre‐pregnancy weight, gestational weight gain, mode of delivery, and gestational age at delivery) and items covering BF practices (skin‐to‐skin contact immediately after birth, early initiation of BF within the first hour after birth, exclusive BF at 6 months, and BF continuation at 1 year and 2 years). Distinguishing exclusive breastfeeding at 6 months from breastfeeding continuation at 12–24 months is conceptually important because these time points reflect different physiological demands and caregiving contexts. Exclusive breastfeeding at 6 months typically coincides with the World Health Organization recommendation (World Health Organization 2017) based on the child's physiological needs, whereas breastfeeding continuation beyond this time point occurs alongside complementary feeding and may reflect cumulative caregiving load, role strain, and sociocultural pressures (Grattan et al. 2024). Examining these exposures separately therefore allows for a more precise assessment of how distinct phases of lactation and caregiving intensity may differentially relate to maternal mental health outcomes.

The second section of the questionnaire included the following validated measures:

#### 2.3.1 Patient Health Questionnaire (PHQ‐4)

The PHQ‐4 is a validated 4‐item screening tool for psychological distress that assesses the frequency of symptoms over the past 4 weeks (Löwe et al. 2010). Each item is rated on a scale from 0 (not at all) to 3 (nearly every day), yielding a total score of 0–12. Two subscales, anxiety and depression, are calculated (0–6 each), with scores ≥ 3 on either subscale considered screen‐positive.

#### 2.3.2 The Women's Health Initiative Insomnia Rating Scale (WHIIRS)

This 5‐item tool evaluates insomnia symptoms based on sleep quality and the frequency of specific sleep disturbances experienced over the past 4 weeks. Total scores range from 0 to 20, with scores ≥ 9 indicating insomnia. While initially designed for postmenopausal women (Levine et al. 2003), it has been used to measure sleep problems during pregnancy, demonstrating good internal consistency with a Cronbach's alpha of 0.77 (Kızılırmak et al. 2012).

#### 2.3.3 The Mediterranean Diet Adherence Screener (MEDAS)

This 14‐item instrument assesses dietary patterns through questions about food intake frequency and consumption of specific food ingredients (Martínez‐González et al. 2012). Responses consistent with Mediterranean diet recommendations score one point, while other responses score 0. Total scores range between 0 and 14, with higher values reflecting higher adherence to the Mediterranean diet. The MEDAS is widely used as a dietary screening tool across diverse populations (Bekar and Goktas 2023; García‐Conesa et al. 2020).

#### 2.3.4 The Breastfeeding Food Security Assessment Scale (BFSAS)

The BFSAS is a 7‐item tool developed to assess food security among breastfeeding women in Lebanon. Items were adapted from the Household Food Insecurity Experience Scale (HFIES) and the Household Food Insecurity Access Scale (HFIAS) and adapted to the breastfeeding context. The scale captures two dimensions of insecurity: concerns about food quality and adequacy of food quantity. Validation analyses demonstrated high internal consistency (Cronbach's *α* = 0.95). Responses are scored on a four‐point frequency scale (no, rarely, sometimes, often), with total scores ranging from 0 to 30. Higher scores indicate more severe food insecurity.

### 2.4 Minimal Sample Size Calculation

The minimum sample size was calculated using G*Power version 3.1.9.7 for Windows (Heinrich Heine, Universität Düsseldorf, Düsseldorf, Germany). Multivariable regressions were planned to identify the correlates of the mental health and insomnia outcomes. Assuming a calculated effect size of f2 = 0.11 (small effect size) and an anticipated squared multiple correlation of 0.1 (R2 deviation from 0) for the Omnibus test of the multiple regression, a minimum of 205 participants was needed, considering an alpha error of 5%, a power of 80%, and 20 predictors to be included in the model. A target of 300 participants was set to account for potential stratification analyses.

### 2.5 Statistical Analysis

Data were analyzed using IBM SPSS Statistics version 25. Descriptive statistics (means, standard deviations, and frequencies) were calculated to summarize sociodemographic, lifestyle, BF practices, and mental health variables. Binary logistic regression analyses were performed to assess the factors associated with the presence of maternal anxiety, depression, and insomnia, defined by PHQ‐4 subscale scores ≥ 3 for anxiety and depression and WHIIRS scores ≥ 9 for insomnia. Independent variables were selected a priori based on theoretical relevance and previous literature. Variables significantly associated with each outcome in bivariate analyses (*p* < 0.05) were entered into the multivariable models.

In the first step, selected variables were entered simultaneously using the forced‐entry (Enter) method. Variables entered into the models included sociodemographic factors (e.g., number of children, smoking, and alcohol use), BF‐related practices (e.g., skin‐to‐skin contact and BF continuation at 6 and 12 months), and psychosocial variables (e.g., insomnia, anxiety, depression, food insecurity, and diet quality based on MEDAS). In the second step, a forward Wald procedure was applied to obtain the final model. Multicollinearity was assessed using Variance Inflation Factor (VIF) and tolerance statistics prior to regression analyses. All VIF values were < 2.5. Adjusted odds ratios (aOR) and 95% confidence intervals (CIs) were reported, and statistical significance was set at *p* < 0.05. Note that missing data was not allowed through the data collection platform and was not replaced.

### 2.6 Ethical Considerations

The study was approved by the Lebanese American University Institutional Review Board (LAU‐IRB; approval number: LAU.SAS.RR2.30/Nov/2023). Electronic informed consent was obtained from all participants via the first page of the Google Form, which presented seven screening and consent statements. Only participants who affirmed all required statements could proceed to the survey.

## 3 Results

The sample comprised 305 mothers with a mean age of 31.97 years. Nearly half (46.9%) had one child, 39.7% had two children, and 13.4% had three or more. Geographically, participants were primarily from Mount Lebanon (44.6%) and Beirut (23.3%). Most respondents held a university degree (86.2%), and 38.4% reported a high income; the mean household crowding index was 1.03. In terms of employment, 19.7% worked in scientific or health fields, 20.3% were housewives, and 21.3% had stopped working to care for their children. During the BF period, 56.1% remained employed. Cesarean section was the predominant mode of delivery (58.4%) (Table 1).

**TABLE 1:** Sociodemographic characteristics of the studied sample.

	*N* (%)
Place of residence most of the year	
Beirut	71 (23.2%)
Mount Lebanon	136 (44.6%)
North	31 (10.2%)
South	42 (13.8%)
Beqaa	25 (8.2%)
Educational level	
School/Technical	42 (13.8%)
University	263 (86.2%)
Total monthly household income	
No income	8 (2.6%)
Low	65 (21.3%)
Intermediate	115 (37.7%)
High	117 (38.4%)
Cigarette smoking	
I have never smoked cigarettes	252 (82.6%)
I am a previous cigarette smoker	28 (9.2%)
I smoke 10 cigarettes or less per day	13 (4.3%)
I smoke 11–20 cigarettes/day	4 (1.3%)
I smoke 20–30 cigarettes/day	5 (1.6%)
I smoke 31 cigarettes and more per day	3 (1.0%)
Waterpipe smoking	
I have never smoked waterpipe	178 (58.4%)
I am a previous waterpipe smoker	46 (15.1%)
I smoke less than 1/week	37 (12.1%)
I smoke 1–2 waterpipes/week	14 (4.6%)
I smoke 2–6 waterpipes/week	17 (5.6%)
I smoke 7 waterpipes and more per week	13 (4.2%)
Alcohol consumption	
No	224 (73.4%)
Yes, previously	30 (9.8%)
Yes, currently	51 (16.8%)
Current occupation	
Employed/Freelancer/Contractor in a scientific or health field	60 (19.7%)
Employed/Freelancer/Contractor in another field	70 (23.0%)
Self‐employed in a scientific or health field	17 (5.6%)
Self‐employed in another field	27 (8.9%)
Student/Trainee in a scientific or health field	2 (0.7%)
Student/Trainee in another field	2 (0.7%)
Housewife (I have never worked)	62 (20.3%)
I stopped working to take care of my children	65 (21.1%)
Occupation during breastfeeding	
Unemployed	134 (43.9%)
Employed	171 (56.1%)
Mode of delivery	
Normal vaginal delivery	127 (41.6%)
Cesarean section	178 (58.4%)
Number of living children	
One	143 (46.9%)
Two	121 (39.7%)
Three or more	41 (13.4%)

	Mean ± SD
Age	31.97 ± 4.774
Household crowding index	1.03 ± 0.46
Duration in months of breastfeeding	11.49 ± 8.99
MEDAS^a^	6.55 ± 1.91
BFSAS^b^	1.64 ± 4.12
WHIIRS^c^	12.96 ± 4.77
Depression^d^	
Yes (PHQ4 Depression ≥ 3)	103 (33.8%)
Anxiety	
Yes (PHQ4 Anxiety ≥ 3)	156 (51.1%)

^a^ MEDAS indicates the Mediterranean diet adherence screener.

^b^ BFSAS indicates the breastfeeding food security assessment scale.

^c^ WHIIRS indicates the women's health initiative insomnia rating scale.

^d^ PHQ4 indicates the patient health questionnaire from which subscales of depression and anxiety are calculated.

Regarding health behaviors, the majority had never smoked cigarettes (82.6%) or waterpipes (58.4%), and 73.4% reported no alcohol consumption. The mean MEDAS score was 6.55 (SD = 1.91), indicating moderate adherence to the Mediterranean diet. The mean duration of BF was 11.49 months, with a mean BFSAS score of 1.64 (SD = 4.12). The mean WHIIRS score was 12.96 (SD = 4.77), indicating a moderate level of insomnia symptoms (Table 1).

Moreover, mental health assessment revealed that 33.8% of participants (*n* = 103) screened positive for depressive symptoms and 51.1% (*n* = 156) for anxiety symptoms on the PHQ‐4. Detailed bivariate analyses are presented in Supporting Table (Appendix 1).

### 3.1 Correlation With Insomnia

In the multivariable logistic regression model (adjusted for sociodemographic, obstetric, and breastfeeding‐related variables entered at step 1), three factors were independently associated with insomnia among women. Breastfeeding continuation to 1 year was associated with higher odds of insomnia (aOR = 2.16, 95% CI 1.04–4.46, *p* = 0.038), indicating that women who continued breastfeeding for 1 year had approximately twice the odds of reporting insomnia compared with those who did not. Similarly, clinically relevant anxiety (score ≥ 3) was associated with increased insomnia risk (aOR = 2.42, 95% CI 1.22–4.82, *p* = 0.012), suggesting more than a twofold elevation in odds. In contrast, higher adherence to the Mediterranean diet (MEDAS score) was inversely associated with insomnia (aOR = 0.80, 95% CI 0.67–0.94, *p* = 0.009), meaning that each unit increase in MEDAS score corresponded to approximately a 20% reduction in the odds of insomnia. All reported associations remained statistically significant after adjustment for potential confounders. Other BF variables were not retained in the model (Table 2).

**TABLE 2:** Correlates of Insomnia among a sample of Lebanese women^a^ bsmo.

		95% CI for aOR		
	Adjusted Odds Ratio (aOR)	Lower	Upper	*p*‐Value
BF continuation to 1 year (Yes vs. No)	2.156	1.042	4.459	0.038*
Anxiety (≥ 3 vs. < 3)	2.419	1.215	4.818	0.012*
MEDAS	0.796	0.670	0.944	0.009**

Abbreviation: CI for aOR confidence interval for the adjusted odds ratio.

^a^ Variables entered on step 1 of the logistic regression: Number of children, Number of children under 5 years old, total monthly household income, Work, Mode of delivery, skin‐to‐skin contact immediately after birth, and encouragement of continuation of contact for an hour or more, encouragement of feeding baby whenever and for as long as he wanted, BF continuation to 1 year, anxiety, depression, MEDAS, and BFSAS.

* *p* < 0.05.

** *p* < 0.01.

### 3.2 Correlates of Anxiety

In the adjusted logistic regression model, several factors were independently associated with anxiety among Lebanese mothers after controlling for sociodemographic, obstetric, behavioral, and breastfeeding‐related variables. A greater number of children was associated with higher odds of anxiety (aOR = 1.61, 95% CI 1.12–2.33, *p* = 0.010). Current tobacco use (any type) was also significantly associated with anxiety (aOR = 2.49, 95% CI 1.34–4.62, *p* = 0.004), indicating more than a twofold increase in odds. Conversely, a higher number of pregnancy weeks was inversely associated with anxiety (aOR = 0.89, 95% CI 0.81–0.98, *p* = 0.022), suggesting a protective effect with advancing gestation. Immediate skin‐to‐skin contact after birth was associated with approximately 50% lower odds of anxiety (aOR = 0.50, 95% CI 0.28–0.91, *p* = 0.022). Strong associations were observed for mental health comorbidities: mothers with depressive symptoms (PHQ ≥ 3) had markedly higher odds of anxiety (aOR = 24.15, 95% CI 11.04–52.82, *p* < 0.001), and insomnia (WHIIRS ≥ 9) was also associated with increased anxiety (aOR = 2.36, 95% CI 1.06–5.26, *p* = 0.037). None of the BF related variables were associated with anxiety (Table 3).

**TABLE 3:** Correlates of anxiety among a sample of Lebanese mothers^a^.

	Adjusted Odds ratio (aOR)	95% CI for aOR		*p*‐Value
		Lower	Upper	
Number of children	1.61	1.12	2.33	0.010*
Smoking any type of tobacco (current)^b^	2.49	1.34	4.62	0.004**
Number of weeks of pregnancy	0.89	0.81	0.98	0.022*
Skin to skin contact (Yes/No)	0.50	0.28	0.91	0.022*
Depression (PHQ ≥ 3 vs. < 3)	24.15	11.04	52.82	< 0.001***
Insomnia (WHIIRS ≥ 9 vs. < 9)	2.36	1.06	5.26	0.037*

Abbreviation: CI: confidence interval for the adjusted odds ratio.

^a^ Variables entered on step 1 of the logistic regression: number of children, educational level, place of residence, crowding index, total monthly household income, work, healthcare background, smoking any type of tobacco, number of weeks of pregnancy, mode of delivery, skin‐to‐skin contact immediately after birth, and encouragement of continuation of this contact for an hour or more, initiation of breastfeeding directly after delivery (within the first hour), rooming in immediately after birth, rooming in for 24 h a day at hospital, BF continuation to 1 year, Depression, Insomnia, MEDAS, and BFSAS.

^b^ Indicates any type of smoking such as waterpipe or cigarette.

* *p* < 0.05.

** *p* < 0.01.

*** *p* < 0.001.

### 3.3 Correlates of Depression

In the fully adjusted logistic regression model, several factors were independently associated with depressive symptoms. Alcohol consumption was inversely associated with depression (aOR = 0.38, 95% CI 0.16–0.93, *p* = 0.033), indicating lower odds of depression among women reporting alcohol use. In contrast, receiving non–breastfeeding‐supportive items (e.g., bottles or formula samples) was associated with more than a twofold increase in the odds of depression (aOR = 2.27, 95% CI 1.09–4.65, *p* = 0.028). A very strong association was observed between anxiety (PHQ ≥ 3) and depression (aOR = 20.00, 95% CI 9.30–42.97, *p* < 0.001), suggesting substantial comorbidity. Finally, higher BFSAS scores were positively associated with depression (aOR = 1.21, 95% CI 1.08–1.35, *p* = 0.001), with each unit increase corresponding to an approximately 21% increase in the odds of depressive symptoms. None of the other BF variables were associated with depression (Table 4).

**TABLE 4:** Correlates of depression among Lebanese women^a^.

	Adjusted Odds Ratio (aOR)	95% CI for aOR		*p*‐value
		Lower	Upper	
Alcohol consumption (Yes vs. No)	0.383	0.158	0.927	0.033*
Receiving non‐BF supportive items^b^	2.271	1.094	4.652	0.028*
Anxiety (PHQ ≥ 3 vs. < 3)	19.995	9.304	42.972	< 0.001***
BFSAS	1.206	1.080	1.347	0.001**

Abbreviation: CI: confidence interval for the adjusted odds ratio.

^a^ Variables entered on step 1 of the logistic regression: age, crowding index, total monthly household income, work, smoking any type of tobacco, alcohol consumption, mode of delivery, non‐BF supportive items, BF initiation directly after delivery (within the first hour)?, Rooming in immediately after birth, rooming in 24 h a day at hospital, being informed of baby hunger signs, awareness of health risks of bottle milk by healthcare workers, anxiety, insomnia, MEDAS, and BFSAS.

^b^ This variable refers to the following question: did healthcare workers offer or give you any items that do not support breastfeeding, such as bottles, nipples, or baby formula samples?

* *p* < 0.05.

** *p* < 0.01.

*** *p* < 0.001.

## 4 Discussion

This study examined associations between BF practices and maternal insomnia, anxiety, and depression while accounting for sociodemographic, dietary, and lifestyle factors. Although the sample demonstrated relatively high educational attainment and moderate‐to‐high income, several other stressors were present. The cesarean section rate (58.4%) exceeded WHO recommendations of 10%–15% (World Health Organization 2015). Over half of participants were employed during the breastfeeding period, and waterpipe smoking was prevalent (41.6%). These structural and cultural factors suggest that maternal mental health outcomes are not only shaped by breastfeeding alone.

Across outcomes, exclusive BF at 6 months was not associated with insomnia, anxiety, or depression. Instead, mental health symptoms were more consistently linked to caregiving burden, socioeconomic stressors, dietary patterns, and psychological conditions.

### 4.1 Breastfeeding and Insomnia

Exclusive BF during the first 6 months was not associated with maternal insomnia, supporting evidence that breastfeeding itself is not inherently disruptive to maternal sleep (Astbury et al. 2022; Palagini et al. 2023). Prior work suggests that sleep fragmentation and the frequency of nighttime awakenings—regardless of feeding modality—are more influential determinants of sleep disturbance (Smith and Forrester 2021). Meta‐analytic and synthesis studies similarly report minimal differences in total sleep duration or quality between feeding methods, with breastfeeding sometimes associated with slightly longer nighttime sleep (Manková et al. 2023; Newman et al. 2023; Srimoragot et al. 2022). Longitudinal data further indicate that night feeding frequency, rather than feeding type, predicts shorter nocturnal sleep and lower sleep efficiency (Astbury et al. 2022), and complementary cohort findings suggest breastfeeding may even be associated with reduced insufficient sleep compared to greater early formula use (Ruan et al. 2022).

In contrast, BF continuation beyond 12 months was positively associated with insomnia. This finding may reflect cumulative caregiving demands rather than breastfeeding exposure per se. Extended breastfeeding often coincides with ongoing night awakenings, maternal fatigue, and psychological strain, particularly in sociocultural settings where caregiving responsibilities remain concentrated on mothers (Nabulsi 2011). Cultural expectations to continue BF, especially when systemic support is limited, may intensify maternal stress (Betts et al. 2021; Gutierrez 2022; Nabulsi 2011): Qualitative and mixed‐methods research indicates that mothers who perceive strong pressure to breastfeed—stemming from healthcare messaging, societal expectations, or internalized “breast is best” ideals—report increased stress and anxiety, in part because they feel they must meet an idealized standard despite practical challenges, lack of autonomy, or inadequate support, which can in turn undermine sleep and overall well‐being (Wheeler et al. 2024). In addition, Western studies also identify anxiety as a central contributor to postpartum sleep disturbances (Cox 2025; Miller et al. 2020; Tham et al. 2016), reinforcing the importance of considering maternal psychological status alongside breastfeeding duration.

Consistent with this, anxiety was significantly associated with insomnia in our sample. Although previous research often positions insomnia as a predictor of anxiety (Osnes et al. 2019; Swanson et al. 2020), the cross‐sectional design prevents determination of temporality. Instead, the findings likely reflect a bidirectional relationship between sleep disturbance and anxiety symptoms.

Skin‐to‐skin contact was not significantly associated with insomnia. While skin‐to‐skin contact promotes bonding and oxytocin release and reduces stress (Association of Women's Health, Obstetric and Neonatal Nurses 2021; Angelhoff et al. 2018; Ionio et al. 2021), its effects may be more immediate and psychological rather than directly influencing long‐term sleep outcomes(Bigelow and Power 2020). Moreover, caregiving demands and nighttime disruptions may persist regardless of early contact practices, particularly in environments lacking shared caregiving responsibilities (Cooijmans et al. 2022).

Dietary patterns also contributed to sleep outcomes. Lower adherence to the Mediterranean diet (MEDAS) was associated with insomnia. Early evidence linked Mediterranean‐style eating to improved sleep and mood regulation (Sánchez‐Villegas et al. 2009), and more recent research has reported fewer sleep disturbances among breastfeeding mothers with higher Mediterranean diet adherence (Tucker and O'Malley 2022). Anti‐inflammatory and nutrient‐rich dietary components may support hormonal balance and stress reduction, thereby benefiting sleep (Kalogerakou and Antoniadou 2024).

Food insecurity (BFSAS) was not associated with insomnia in this study, although prior research links maternal food insecurity to anxiety and depressive symptoms (Shreffler et al. 2024; Tarasuk et al. 2020), both established contributors to sleep disturbance. This discrepancy may reflect sample characteristics or overlapping adjustment for mental health symptoms.

Overall, the insomnia findings suggest that caregiving burden, anxiety, cultural expectations, and modifiable lifestyle factors are more central determinants of maternal sleep than exclusive breastfeeding itself.

### 4.2 Breastfeeding and Anxiety

No significant association was found between BF duration and maternal anxiety, consistent with prior evidence (Hoff et al. 2019). A recent meta‐analysis indicates that breastfeeding is associated with improved maternal mental health overall; however, outcomes depend heavily on the quality of the breastfeeding experience (Yuen et al. 2022). When mothers encounter difficulties or perceive misalignment between expectations and experience, anxiety may increase. Thus, breastfeeding duration alone may be an insufficient indicator of maternal psychological outcomes (Yuen et al. 2022).

Skin‐to‐skin contact emerged as a protective factor against anxiety, consistent with literature highlighting its role in enhancing maternal–infant bonding and reducing stress via oxytocin‐mediated mechanisms (Asimaki et al. 2022; Moore et al. 2016; Yuen et al. 2022). This finding underscores the importance of early relational interventions in supporting maternal emotional well‐being.

Anxiety was also associated with insomnia and depression, reflecting well‐documented comorbidity during the perinatal period (Kalin 2020). Sleep disturbances may impair emotional regulation and heighten stress vulnerability (Cox 2025; Schantz et al. 2024), potentially mediated by neuroendocrine pathways involving cortisol and oxytocin dysregulation (Dressle et al. 2022; Raymond et al. 2021). Additionally, tobacco use (cigarettes or waterpipes) was significantly associated with anxiety, supporting evidence that smoking may serve as a maladaptive coping strategy while exacerbating psychological symptoms (Alibekova et al. 2016; Hahad et al. 2022; McDermott et al. 2013). In the broader population, smoking may reflect underlying psychosocial stressors or limited social support (Garg et al. 2021).

Collectively, these findings indicate that maternal anxiety is associated with higher psychosocial vulnerability rather than with breastfeeding duration.

### 4.3 Breastfeeding and Depression

BF duration at 6 and 12 months was not associated with maternal depression, consistent with some previous studies (Mikšić et al. 2020; Nagel et al. 2022). However, literature remains mixed. Some research reports protective effects of breastfeeding on depressive symptoms, potentially mediated by oxytocin pathways (Dias and Figueiredo 2015), and a recent meta‐analysis found lower PPD among women who exclusively breastfed (Xia et al. 2022). The relationship may be bidirectional: PPD can undermine breastfeeding confidence and continuation, potentially worsening depressive symptoms in a self‐reinforcing cycle (Ahmadinezhad et al. 2024; Dias and Figueiredo 2015; Palancı and Aktaş 2024). The cross‐sectional design of the present study precludes clarification of these temporal dynamics.

Anxiety was the strongest correlate of depression, consistent with evidence of substantial comorbidity in the perinatal period (Ou et al. 2025; Shen et al. 2024). These findings reinforce recommendations for integrated screening and management of anxiety and depression in postpartum care (ACOG 2023a, 2023b).

Exposure to non‐BF supportive items provided by healthcare workers was associated with higher odds of depression. Prior work suggests that formula samples and pacifiers may influence breastfeeding behaviors and potentially undermine maternal confidence (Batista et al. 2018), while unsupportive hospital practices may negatively affect maternal emotional well‐being (McLeish et al. 2021).

Greater food insecurity (BFSAS) was also associated with depression, consistent with research linking inadequate food access to maternal psychological distress (Natamba et al. 2017; Reesor‐Oyer et al. 2021; Subramani 2024). This highlights the intersection between socioeconomic hardship and postpartum mental health.

Alcohol consumption was inversely associated with depression in this sample. One plausible explanation is that light or moderate alcohol consumption may serve as a social or coping mechanism that reduces perceived stress or improves mood in the short term, which could attenuate depressive symptoms in some postpartum women (Qiu et al. 2022). However, this association may also reflect confounding by social support, socioeconomic status, or health behaviors, rather than a causal protective effect of alcohol itself. Similar inverse associations between moderate alcohol use and lower depression scores have been observed in population studies, though they are often attenuated after adjusting for confounders (Boden and Fergusson 2011). Nevertheless, alcohol is generally recognized as a risk factor for mental health problems, and contextual factors may explain this association (Hubbard and Falco 2015). Caution is therefore warranted in interpreting this finding. Finally, structural and psychosocial determinants, including anxiety, food insecurity, and perceived breastfeeding support, demonstrated stronger associations with maternal depression than breastfeeding duration itself (Yim et al. 2015).

### 4.4 Strengths and Limitations

This study provides valuable insights into breastfeeding practices and maternal mental health in Lebanon, using recent data, validated tools, and controlling for sociodemographic, dietary, and lifestyle factors. However, some limitations must be acknowledged. The cross‐sectional design precludes causal inference, and the use of a convenience non‐random sample may introduce selection bias and limit generalizability, despite the efforts to optimize representativity. Self‐reported data on breastfeeding practices, such as skin‐to‐skin contact and exclusive BF at 6 months or 12 months, as well as mental health, are subject to recall, information, and social desirability bias. Moreover, the PHQ‐4 is a screening rather than a diagnostic tool and has some degree of construct overlap between anxiety and depressive symptoms, while WHIIRS has population specificity among broader adult samples, limiting its generalizability in younger or more diverse populations. Finally, the postpartum duration varied among women and was not controlled for in our analysis. In addition, the lack of differentiation by feeding intensity, nighttime feeding frequency, mixed feeding, and pumping practices, all of which are particularly relevant to sleep‐related outcomes (Srimoragot et al. 2022) and may contribute to exposure misclassification, might affect our findings. Furthermore, cultural and social pressures surrounding breastfeeding in Lebanon were not directly measured or analytically integrated (Osman et al. 2009): these may contribute to residual confounding, and their effect could change the magnitude of the detected associations between breastfeeding and mental health components. Dedicated studies that detail the feeding practices and cultural/social aspects of breastfeeding are suggested to further confirm and refine our results.

## 5 Conclusion

This study highlights the multifactorial associations between breastfeeding practices and mental health among Lebanese mothers. While exclusive breastfeeding during the first 6 months was not linked to maternal insomnia, anxiety, or depression, prolonged breastfeeding beyond 12 months was associated with increased insomnia. Exploratory findings showed that adherence to the Mediterranean diet and skin‐to‐skin contact improved maternal mental health. Future longitudinal research is needed to clarify causal pathways and evaluate interventions tailored to Lebanon's unique sociocultural context. These will help in developing breastfeeding promotion programs integrating key elements such as mental health screening, which may improve maternal and offspring outcomes.

## Author Contributions

**Hala Sacre:** writing – original draft, conceptualization, writing – review and editing, data curation. **Joanne Karam:** methodology, data curation, writing – review and editing. **Chadia Haddad:** data curation, methodology, writing – review and editing. **Cosette Fakih El Khoury:** methodology, writing – review and editing, data curation. **Paula Hage Boutros:** conceptualization, methodology, data curation, writing – review and editing, formal analysis, software, validation, writing – original draft. **Bahia Abdallah:** methodology, conceptualization, writing – review and editing, data curation, validation. **Pascale Salameh:** conceptualization, methodology, writing – review and editing, data curation. **Nour Chamma:** writing – review and editing, writing – original draft, conceptualization. **Rana Rizk:** conceptualization, methodology, writing – review and editing, data curation, validation.

## Funding

The authors have nothing to report.

## Ethics Statement

The study received ethical approval by the Lebanese American University‐ Institutional Review Board (LAU‐IRB) (LAU.SAS.RR2.30/Nov/2023). Before completing the questionnaire, all participants provided their consent by clicking on the respective button on the first page of the Google Form.

## Conflicts of Interest

The authors declare no conflicts of interest.

## Supporting information


**Appendix 1.** Supplementary Table: Bivariate Associations between independent variables and mental health outcomes (insomnia, depression, anxiety).

## Data Availability

The data that support the findings of this study are available on request from the corresponding author. The data are not publicly available due to privacy or ethical restrictions.
