# Supplementary material for: Breastfeeding Practices, Insomnia Symptoms, Anxiety, and Depression Among Lebanese Mothers: A Cross‐Sectional Study
Source: Food Sci Nutr. 2026 May 22;14(5):e71830. doi: 10.1002/fsn3.71830 (PMC13239503; doi:10.1002/fsn3.71830)
Supplement: Supplementary file 1 — Appendix 1. Supplementary Table: Bivariate Associations between independent variables and mental health outcomes (insomnia, depression, anxiety). [file FSN3-14-e71830-s001.docx]

| **Supplementary Table: Bivariate Associations Between Independent Variables and Mental Health Outcomes (Insomnia, Depression, Anxiety)** | | | | | | |
| --- | --- | --- | --- | --- | --- | --- |
|  | **Depression**  **(PHQ-4 depression subscale)** | **p-value** | **Anxiety**  **(PHQ-4 anxiety subscale)** | **p-value** | **Insomnia Scale (WHIIRS)** | **p-value** |
|  | **Mean ± SD** |  | **Mean ± SD** |  | **Mean ± SD** |  |
| **Educational level** |  | 0.100 |  | 0.106 |  | **0.008** |
| School/Technical | 2.78±1.76 |  | 3.42±1.50 |  | 14.64±4.14 |  |
| University | 2.29±1.78 |  | 2.94±1.83 |  | 12.69±4.81 |  |
| **Total monthly household income** |  | **<0.001** |  | **<0.001** |  | **<0.001** |
| None | 2.75±1.98 |  | 3.12±1.88 |  | 13.75 ±4.77 |  |
| Low | 3.27±1.92 |  | 3.98±1.66 |  | 14.95±4.15 |  |
| Intermediate | 2.46±1.74 |  | 3.00±1.83 |  | 12.89±4.96 |  |
| High | 1.73±1.49 |  | 2.47±1.61 |  | 11.87±4.57 |  |
| **Cigarette smoking**  Nonsmoker/ex-smoker  Active smoker  **Waterpipe smoking**  Nonsmoker/ex-smoker  Active smoker  **Alcohol consumption**  No/ previous  Yes, currently  **Current occupation**  Employed  Unemployed | 2.28±1.77  3.20±1.80  2.25±1.80  2.66±1.71  2.45±1.81  1.90±1.57  2.20±1.65  2.57±1.93 | **0.015**  0.076  **0.029**  0.084 | 2.94±1.77  3.80±1.84  2.84±1.81  3.48±1.65  3.07±1.76  2.68±1.90  2.82±1.75  3.25±1.82 | **0.034**  **0.006**  0.155  **0.037** | 12.85±4.76  14.16±4.74  12.62±4.86  13.88±4.38  13.08±4.88  12.37±4.13  12.38±4.82  13.73±4.59 | 0.191  **0.041**  0.333  **0.014** |
| **Breastfeeding of the last child**  No  Yes | 3.36±0.64  2.33±0.10 | 0.059 | 3.55±0.58  2.99±0.10 | 0.317 | 9.55±2.04  13.09±0.27 | 0.115 |
| **Occupation during breastfeeding** |  | **0.005** |  | **0.006** |  | **0.010** |
| Employed | 2.10±1.61 |  | 2.76±1.72 |  | 12.34±4.87 |  |
| Unemployed | 2.69±1.94 |  | 3.32±1.84 |  | 13.75±4.52 |  |
| **Mode of delivery of the last child** |  | 0.150 |  | **0.003** |  | **0.012** |
| Normal vaginal delivery | 2.18±1.74 |  | 2.65±1.77 |  | 12.13±5.12 |  |
| Cesarean section | 2.48±1.81 |  | 3.26±1.76 |  | 13.55±4.41 |  |
|  | **Correlation coefficient** | **p-value** | **Correlation coefficient** | **p-value** | **Correlation coefficient** | **p-value** |
| **Age in years** | -0.163 | **0.004** | -0.081 | 0.159 | -0.068 | 0.235 |
| **Duration of breastfeeding** | 0.067 | 0.245 | 0.023 | 0.694 | 0.111 | 0.053 |
| **Household crowding index** | 0.260 | **<0.001** | 0.305 | **<0.001** | 0.171 | **0.003** |
| **Number of live children**  **MEDAS scale** | 0.033  -0.268 | 0.560  **<0.001** | 0.158  -0.269 | **0.006**  **<0.001** | 0.042  -0.331 | 0.468  **<0.001** |
| **Anxiety scale** | 0.752 | **<0.001** | - | **-** | 0.323 | <0.001 |
| **Insomnia scale** | 0.276 | **<0.001** | 0.323 | **<0.001** | - | - |
